# Supplementary material for: Non-Markovian relaxation spectroscopy of fluxonium qubits
Source: Nat Commun. 2026 Feb 24;17:3209. doi: 10.1038/s41467-026-69910-2 (PMC13057262; doi:10.1038/s41467-026-69910-2)
Supplement: Supplementary file 1 — Supplementary Information [file 41467_2026_69910_MOESM1_ESM.pdf]

Supplementary Material for "Non-Markovian Relaxation Spectroscopy of a Fluxonium Qubit"

Contents

I. Measurement setup 2

II. Standard T_1 measurements are susceptible to misinterpretation 3

III. Modeling two-timescale relaxometry 4
- A. Decay rate extraction in Markovian environment 4
- B. TLS polarizability in Non-Markovian environment 5
- C. Decay rate evolution with changing environment 7
- D. Choice of protocol parameters and quasi-steady state approximation 9
- E. Verification of methods via full dynamics simulation 11

IV. Calibration experiments 12

V. Two-timescale relaxometry with variable polarity-switch structure 16

VI. Surface participation ratio (SPR) simulation and TLS electric dipole moment 20
- A. SPR simulation 20
- B. TLS dipole moment estimation 21

References 22

I Measurement setup

Both experiments are carried out in an Oxford Triton 500 Dilution Refrigerator. For the 3D device, We use an amuneal can to protect the device from unwanted external magnetic field. To shield the device from IR radiation, homemade microwave absorber (consists of Stycast 1266, Carbon Black and Silicon Carbide) is painted inside the can and we use eccosorb foam covering inside wall of the can. The whole device is positioned under mixing chamber plate (MXC) that stands a base temperature of around 20 mK. As shown in Fig S1 (a), on the input line, we adopt 70 dB attenuators (40 dB under MXC), a Marki 9.6 GHz low pass filter and three homemade eccosorb (one of them inside the can). For the output line, we apply two homemade eccosorb (one of them inside the can), a Marki 7.85 GHz bandpass filter, a single junction isolator and a double junction isolator to avoid microwave noise entering from output line, followed by a High-Electron-Mobility Transistor (HEMT) amplifier at the 4K stage and a room temperature amplifier. Both RF lines have DC blocks in room temperature setup. We use a DC source (YOKOGAWA 7651) applying current on a superconducting coil to tune flux for the fluxonium qubit. As for the 2D device (Fig. S1 (b)), we use one RF System-on-Chip board (Quantum Instrumentation Control Kit, ZCU216) for qubit control and readout. There are some major changes inside the fridge: fluxonium drive line is separated, with 60 dB attenuation inside fridge; resonator drive line also has 60 dB attenuation; both input lines have a low pass filter and one eccosorb; no absorber or eccosorb foam is used.

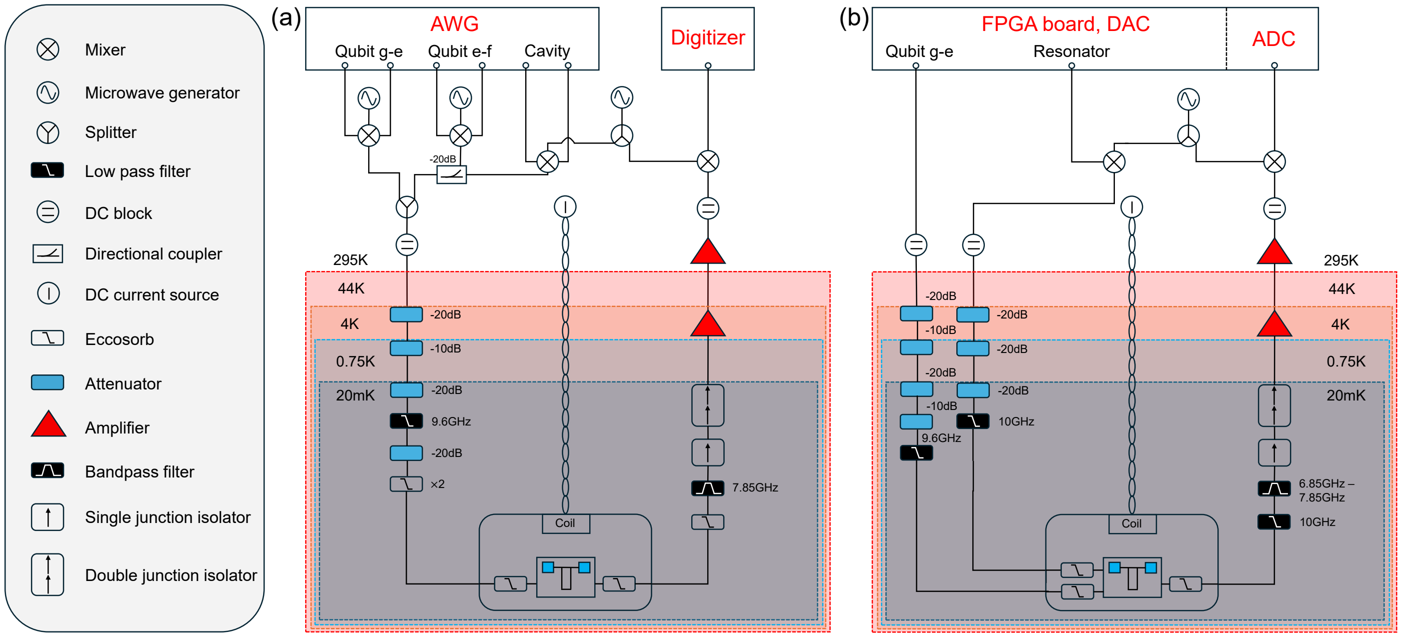

FIG. S1. **Cryogenic and room temperature experimental microwave setup. (a)** 3D device. **(b)** 2D device.

II Standard T_1 measurements are susceptible to misinterpretation

In this section, we use some simulated data to illustrate how non-Markovian relaxation dynamics can go unnoticed in a traditional T_1 measurement. The standard exponential fits to decay curves, especially in the presence of noise, and leads to misinterpreted qubit lifetimes that depends on the details of the protocol. Here we simulate four common scenarios to measure the qubit T_1 when a long-lived TLS is unknowingly affecting the qubit relaxation dynamics. The four scenarios include a combination of two qubit-reset methods (clock cycle and active reset) and two average methods (rounds and repetitions). For clock cycle, we prepare qubit with a qubit $|g\rangle - |e\rangle$ π pulse ($\pi_{\rm ge}$) and measure after a variety of delay times, but we keep same cycle period between two neighboring $\pi_{\rm ge}$ pulses. Active reset, on the other hand, gives very short delay after the measurement as we

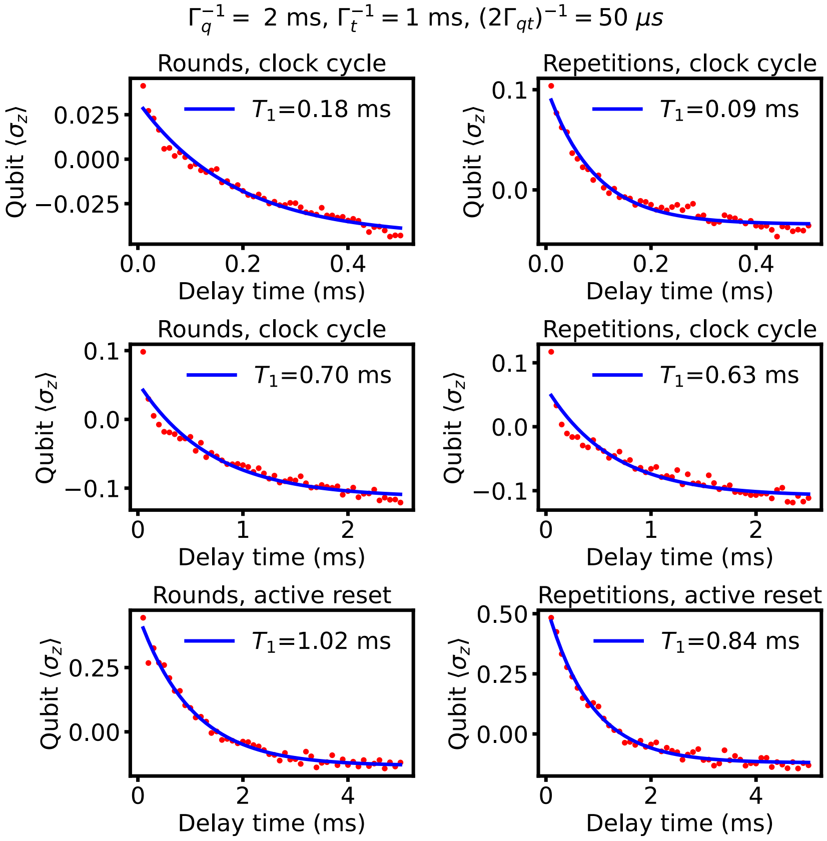

FIG. S2. **Various T_1 protocols suffer TLS's impact.** Red dots represents data simulated by assigned qubit and TLS parameters together with experiment protocols, and a gaussian-distributed noise is added to the dataset. Blue line is a single exponential fitting to the dataset and so-called "T_1" results are listed. In the left line all measurements are done with rounds and for the right line they are done in repetitions. For the top two figures, we assume the step is 10 µs and for the middle two figures it is 50 µs. All four plots are done with clock cycle protocol. As for the bottom two figures, we apply active reset protocol and the step is 100 µs.

assume there is a robust way to reset the qubit to the same state. Any reset strategy may fall into this category if we don't realize it can only reset the qubit but not the environment. "Rounds" repeatedly cycles through all measurements by measuring once at each delay time, while "repetitions" means repeating and averaging measurement results at one delay time before moving to the next.

In the simulation we assume $\Gamma_{\mathrm{qt}} = 10/\mathrm{ms}$, which leads to a relaxation timescale of 50 µs. $Z^{\mathrm{eq}} = -0.2$, $p_{\mathrm{t}}^{\mathrm{eq}} = -0.1$, and the active reset process can always bring qubit Z_{e} to 0.5. For each trace we give 50 data points. The readout pulse length is set to be 15 µs and the process is assumed to be quantum non-demolition, so that each readout result represents average qubit population during the readout period. The cycle time for the top two figures is 0.5 ms and is 2.5 ms for the middle two ones, while the relax time after measurement for the active reset protocol is 10 µs. $\Gamma_{\mathrm{q}} = 0.5/\mathrm{ms}$ and $\Gamma_{\mathrm{t}} = 1/\mathrm{ms}$. Reasonable amount of noise is added to all the data points. From our simulation results (Fig. S2), T_1 measurement by reset method without enough relax delay is very likely to hide non-Markovianity as measured T_1 cannot reflect Γ_{q}, Γ_{t} or Γ_{qt} and it does look like an exponential feature. As for clock cycle protocol, we note that there are also many deceptive results but maybe recognizable if pay attention to short-delay points, as well as the whole contrast value of the curve, which can be much smaller if cycle period or relax delay is not enough. But it is still hard to extract multiple time scales from these protocols without rough estimation of TLS lifetime. We note that fixed delay method, another frequently-used protocol that fixes the time between measurement and next qubit preparation π_{ge} pulse, shares similar features as clock cycle protocol with our assigned parameters. Overall, our simulation results show that normal T_1 measurement protocols can easily cover non-Markovianity and lead to misinterpretation of qubit relaxation dynamics, which needs to be corrected by our proposed method.

III Modeling two-timescale relaxometry

A. Decay rate extraction in Markovian environment

To introduce concepts in our model, we start from assuming the environment is Markovian. In this case, qubit decay rates $\Gamma_{\downarrow}$ and $\Gamma_{\uparrow}$ are independent of measurement history. Define $Z \equiv \langle \sigma_{\mathrm{z}} \rangle$ for qubit. When qubit is perfectly initialized in $|e\rangle$ state, we note $Z_{\mathrm{e}}^{\mathrm{ideal}} = 1$ and accordingly, $Z_{\mathrm{g}}^{\mathrm{ideal}} = -1$.

Qubit decay slope $\frac{dZ}{dt}$ depends on the qubit polarization as follow:

$$\frac{dZ}{dt} = -(1+Z)\Gamma_{\downarrow} + (1-Z)\Gamma_{\uparrow}. \tag{S1}$$

When reaching equilibrium, the qubit equilibrium polarization Z^{eq} can be expressed by $\Gamma_\uparrow$ and $\Gamma_\downarrow$:

$$\frac{dZ}{dt} = 0 \Rightarrow Z^{\text{eq}} = -\frac{\Gamma_\downarrow - \Gamma_\uparrow}{\Gamma_\downarrow + \Gamma_\uparrow}. \tag{S2}$$

Define Γ_Σ and Γ_δ:

$$\begin{aligned} \Gamma_\Sigma &\equiv \Gamma_\downarrow + \Gamma_\uparrow, \\ \Gamma_\delta &\equiv \Gamma_\downarrow - \Gamma_\uparrow. \end{aligned} \tag{S3}$$

So that: $Z^{\text{eq}} = -\frac{\Gamma_\delta}{\Gamma_\Sigma}$. Now we can rewrite the qubit decay slope (transition rate) in terms of Γ_δ and Γ_Σ:

$$\frac{dZ}{dt} = -Z\Gamma_\Sigma - \Gamma_\delta. \tag{S4}$$

Assume we can initialize our qubit towards two different states, "excited" and "ground", with $Z = Z_{\text{e}}$ and $Z = Z_{\text{g}}$, respectively. Qubit state preparation is not ideal in practice, but in general, $Z_{\text{e}} > 0$ and $Z_{\text{g}} < 0$. We prepare each state, apply different delay times t_i, and perform a single-exponential fit to the measured readout populations Z_i to extract the initial decay slope:

$$Z = b_g - (\frac{dZ}{dt})_0 \tau e^{-t/\tau}, \tag{S5}$$

where b_g and τ are the background and time constant of the exponential fit, respectively.

Now, we have two equations to solve two unknowns $(\Gamma_\Sigma, \Gamma_\delta)$:

$$\begin{aligned} (\tfrac{dZ}{dt})_{0,\text{e}} &= -Z_{\text{e}}\Gamma_\Sigma - \Gamma_\delta, \\ (\tfrac{dZ}{dt})_{0,\text{g}} &= -Z_{\text{g}}\Gamma_\Sigma - \Gamma_\delta. \end{aligned} \tag{S6}$$

B. TLS polarizability in Non-Markovian environment

Now we assume that the environment depends on a specific measurement sequence. We introduce a new time axis, T, referring to this changing environment, which is different from delay time t in normal T_1 measurement. Qubit relaxation and excitation rates can now in some way depend on this variable T. $\Gamma_\downarrow, \Gamma_\uparrow \Rightarrow \Gamma_\downarrow(T), \Gamma_\uparrow(T)$. All other equations in sub-section III A hold true in this sub-section.

We consider numbers of TLS with frequency of $\omega_{\text{t},k}$, where k represents the kth TLS. We define that $\Gamma_{\text{q}} \equiv \Gamma_{\downarrow,q} + \Gamma_{\uparrow,q}$ and $\Gamma_{\text{t},k} \equiv \Gamma_{\downarrow,k} + \Gamma_{\uparrow,k}$ are the intrinsic qubit and TLS decay rates, respectively. They are independent from each other and other parameters. $\Gamma_{\text{qt},k}$ is the qubit-TLS interaction rate, which is a function of the qubit frequency ω_{q} [S1]:

$$\Gamma_{\text{qt},k}(\omega_{\text{q}}) = \frac{2g_k^2\Gamma_{2,k}}{{\Gamma_{2,k}}^2 + (\omega_{\text{t},k} - \omega_{\text{q}})^2}, \tag{S7}$$

where g_k is the coupling rate between qubit and k^{th} TLS, and $\Gamma_{2,k} = \frac{\Gamma_{\text{q}}}{2} + \frac{\Gamma_{\text{t},k}}{2} + \Gamma_{\phi,\text{q}} + \Gamma_{\phi,k}$ is the total decoherence rate of the qubit and k^{th} TLS (in our case $\Gamma_{\text{q}}, \Gamma_{\text{t},k}, \Gamma_{\phi,\text{q}} \ll \Gamma_{\phi,k}$).

Assume that Z and p_k denote the polarizations of the qubit and the kth TLS, respectively, while Z^{eq} and p_k^{eq} represent their corresponding thermal equilibrium polarizations. We then write down the decay slope equations for polarizations of both qubit and TLS as functions of qubit and TLS states [S2–S5]:

$$\frac{dZ}{dt} = -\Gamma_{\text{q}}(Z - Z^{\text{eq}}) - \sum_k \Gamma_{\text{qt},k}(Z - p_k), \tag{S8}$$

$$\frac{dp_k}{dt} = -\Gamma_{\text{t},k}(p_k - p_k^{\text{eq}}) - \Gamma_{\text{qt},k}(p_k - Z). \tag{S9}$$

Each TLS's quasi-steady-state polarization p_k' is found by setting its time derivative $\dot{p}_k$ to 0:

$$p_k' = \frac{\Gamma_{\text{t},k} p_k^{\text{eq}} + \Gamma_{\text{qt},k} Z}{\Gamma_{\text{t},k} + \Gamma_{\text{qt},k}}. \tag{S10}$$

For our experiment protocol, the polarization sequence consists of constantly initializing the qubit to one of two known states (one towards $|e\rangle$ and the other close to $|g\rangle$), followed by readout measurements after 4 delay times to extract qubit decay rates. During the measurement sequence, the qubit-TLS interaction is not always on: during qubit initialization and readout, as the qubit frequency is shifted by $\sim$40 MHz, we believe the qubit is decoupled from the environment we are probing, i.e. $\Gamma_{\text{qt}}^{\text{eff}} = 0$. The fraction of time during which the interaction is fully active (i.e., equal to Γ_{qt}) is represented by an efficiency factor η. Due to qubit decay during the delay times, the qubit polarization varies during the measurement sequence, and the time-averaged polarization of the qubit during the sequence is the important quantity. When the TLS bath is fully polarized, two halves of the measurement sequence (one to polarize the TLS up, and the other to polarize it down) have different average qubit polarizations ($\bar{Z}_{\text{H}}$ and $\bar{Z}_{\text{L}}$) considering all the qubit polarization dynamic details. The lowest and highest TLS polarizations this protocol can reach are calculated out by plugging these two quantities into Z in Eq. (S10):

$$p_k^{\min} = \frac{\Gamma_{\text{t},k} p_k^{\text{eq}} + \Gamma_{\text{qt},k} \bar{Z}_{\text{L}} \eta}{\Gamma_{\text{t},k} + \Gamma_{\text{qt},k}\eta}, \tag{S11}$$

$$p_k^{\max} = \frac{\Gamma_{\text{t},k} p_k^{\text{eq}} + \Gamma_{\text{qt},k} \bar{Z}_{\text{H}} \eta}{\Gamma_{\text{t},k} + \Gamma_{\text{qt},k}\eta}. \tag{S12}$$

This allows us to define each TLS's polarizability:

$$p_k^{\Delta} \equiv p_k^{\max} - p_k^{\min} = \frac{\Gamma_{\text{qt},k}\eta}{\Gamma_{\text{t},k} + \Gamma_{\text{qt},k}\eta} \left[\bar{Z}_{\text{H}} - \bar{Z}_{\text{L}}\right], \tag{S13}$$

where $\bar{Z}_{\mathrm{H}}$ and $\bar{Z}_{\mathrm{L}}$ are the time-averaged qubit polarizations during the first half (reset towards $|e\rangle$) and the second half (reset towards $|g\rangle$) of the final bath-polarizing cycles. In our experiment, time-averaged qubit polarization is expressed as:

$$\bar{Z} = \frac{\sum_i \left[\int_0^{t_i} b_g - (\frac{dZ}{dt})_0 \tau e^{-t/\tau} \, dt + Z_{\mathrm{g/e}} t_{\mathrm{idle}} \right]}{\sum_i \left[t_i + t_{\mathrm{idle}} \right]}. \tag{S14}$$

$Z_{\mathrm{g/e}}$ is determined by qubit initialization condition (details in Fig. S3 and Section IV), and t_{idle} is the idle time after pumping (15 µs here, and details in Table S1). In the 3D device, $t_i = 1, 40, 150, 330$ µs for FD-4 and $t_i = 1, 10, 20...70$ µs for CD-8. By substituting the corresponding values of b_g, τ, and $\left(\frac{dZ}{dt}\right)_0$ from the final bath-polarizing cycles into the above expression, we obtain $\bar{Z}_{\mathrm{H}}$ and $\bar{Z}_{\mathrm{L}}$.

C. Decay rate evolution with changing environment

So far we have derived how much difference we can make to the TLS polarization with our protocol, and the TLS state change can lead to qubit decay/excitation rate evolution. The environment bath is affected by the bath-polarizing sequences in the following way: when the qubit interacts with a TLS, repeatedly bringing the qubit to its excited/ground state polarizes the TLS to its own high/low state, respectively. Such polarization effect exists even qubit and TLS are far-detuned. Consider the measurement sequence in two halves. In the first half, the qubit is repeatedly brought to its excited state, and its initial transition rate (mostly consists of $\Gamma_\downarrow(T)$, but due to imperfection in qubit state preparation, part of $\Gamma_\uparrow(T)$ is also included) is measured. Repeated measurements of this type act to polarize the TLS to its excited state (note the highest state it can reach as "H"). As TLS approaches its maximum excited state, $\Gamma_\downarrow(T)$ decreases to its minimum. The second half of the measurement is essentially the reverse action: the qubit is repeatedly brought to its ground state, and its transition rate is again measured. The TLS is pushed towards its ground state (note the lowest state as "L"), and $\Gamma_\uparrow(T)$ decreases.

For the first half of the measurement that polarizes TLS from L to H state, the sequence resets the qubit to $|e\rangle$ and both $\Gamma_\uparrow$ and $\Gamma_\downarrow$ follow an exponential trend, with time constant of τ_{e} (note that in principle there should be a sum here for various timescales, but here we assume single environmental timescale, which can describe our experiment data well, especially capture start and end points):

$$\Gamma_\uparrow = \Gamma_{\uparrow\mathrm{H}} + (\Gamma_{\uparrow\mathrm{L}} - \Gamma_{\uparrow\mathrm{H}}) e^{-T/\tau_{\mathrm{e}}}, \tag{S15}$$

$$\Gamma_\downarrow = \Gamma_{\downarrow\mathrm{H}} + (\Gamma_{\downarrow\mathrm{L}} - \Gamma_{\downarrow\mathrm{H}}) e^{-T/\tau_{\mathrm{e}}}. \tag{S16}$$

Plug these formulas together with (S3) into (S6), the initial slope of the qubit decay can then be written as:

$$\frac{dZ}{dt}(\downarrow) = -Z_{\mathrm{e}}\Gamma_{\Sigma\mathrm{H}} - \Gamma_{\delta\mathrm{H}} - [Z_{\mathrm{e}}(\Gamma_{\Sigma\mathrm{L}} - \Gamma_{\Sigma\mathrm{H}}) + \Gamma_{\delta\mathrm{L}} - \Gamma_{\delta\mathrm{H}}]e^{-T/\tau_{\mathrm{e}}}. \tag{S17}$$

For the second half, the TLS polarization reverses direction, from H to L state. Similarly, the initial slopes are derived to be:

$$\frac{dZ}{dt}(\uparrow) = -Z_{\mathrm{g}}\Gamma_{\Sigma\mathrm{L}} - \Gamma_{\delta\mathrm{L}} - [Z_{\mathrm{g}}(\Gamma_{\Sigma\mathrm{H}} - \Gamma_{\Sigma\mathrm{L}}) + \Gamma_{\delta\mathrm{H}} - \Gamma_{\delta\mathrm{L}}]e^{-T/\tau_{\mathrm{e}}}. \tag{S18}$$

From general observations in our experiments, we find that $\Gamma_{\Sigma\mathrm{H}} = \Gamma_{\Sigma\mathrm{L}}$ is a proper assumption, so that Γ_{Σ} is independent of TLS states. Now we can link Γ_{qt}, Γ_{q} to Γ_{Σ} and Γ_{δ}. Recall equations (S1) and (S8), so that:

$$\Gamma_{\mathrm{q}}(Z - Z^{\mathrm{eq}}) + \sum_k \Gamma_{\mathrm{qt},k}(Z - p_k) = (1+Z)\Gamma_{\downarrow} - (1-Z)\Gamma_{\uparrow}. \tag{S19}$$

Separate terms that contain Z and only constant. By comparing the coefficients, we can now find that:

$$\Gamma_{\Sigma} = \Gamma_{\mathrm{q}} + \sum_k \Gamma_{\mathrm{qt},k}, \tag{S20}$$

$$\Gamma_{\delta} = -\Gamma_{\mathrm{q}}Z^{\mathrm{eq}} - \sum_k \Gamma_{\mathrm{qt},k}p_k. \tag{S21}$$

Γ_{δ} is dependent on TLS polarizations p_k. Now consider the upper and lower limit of Γ_{δ}, which correspond to environment in its L and H states, respectively. At that time Γ_{δ} is obtained by plugging in all TLS's highest and lowest state polarizations $p_k^{\min}$ and $p_k^{\max}$:

$$\Gamma_{\delta\mathrm{L}} = -\Gamma_{\mathrm{q}}Z^{\mathrm{eq}} - \sum_k \Gamma_{\mathrm{qt},k}p_k^{\min}, \tag{S22}$$

$$\Gamma_{\delta\mathrm{H}} = -\Gamma_{\mathrm{q}}Z^{\mathrm{eq}} - \sum_k \Gamma_{\mathrm{qt},k}p_k^{\max}. \tag{S23}$$

So that by applying the polarization sequences in our experiment, the polarizable part of Γ_{δ} rate is:

$$\Gamma_{\delta\delta} = \Gamma_{\delta\mathrm{L}} - \Gamma_{\delta\mathrm{H}} = \sum_k \Gamma_{\mathrm{qt},k}p_k^{\Delta} = \sum_k \frac{\Gamma_{\mathrm{qt},k}^2\eta}{\Gamma_{tk} + \Gamma_{\mathrm{qt},k}\eta}\left[\bar{Z}_{\mathrm{H}} - \bar{Z}_{\mathrm{L}}\right]. \tag{S24}$$

Note that in the FD-4 experiment, we assume qubit initialization states is consistent for a given pumping scheme and qubit frequency, implying that it is independent of TLS state. This is a good approximation when the qubit-TLS interaction is not very strong. For the CD-8 experiment intended for the regime of strong qubit-TLS interaction, we do see qubit initializations vary slightly with TLS state. This is because the TLS affects

the qubit state during the short idling time in our reset protocol. Thus, Z_{e} and Z_{g} are functions of T. Our slope functions become:

$$\begin{aligned}(\tfrac{dZ}{dt})_{0,\mathrm{e}} &= -Z_{\mathrm{e}}(T)\Gamma_{\Sigma} - \Gamma_{\delta},\\ (\tfrac{dZ}{dt})_{0,\mathrm{g}} &= -Z_{\mathrm{g}}(T)\Gamma_{\Sigma} - \Gamma_{\delta}.\end{aligned} \tag{S25}$$

Assume that $Z_{\mathrm{e}}(T)$ and $Z_{\mathrm{g}}(T)$ both evolve exponentially with T, and time constant is the same as τ_{e}. General observation indicates that the contrast of two initializations are equal, so $\delta Z_{\mathrm{e}} = \delta Z_{\mathrm{g}} = \delta Z$. Then the slope functions become:

$$\begin{aligned}\tfrac{dZ}{dt}(\downarrow) &= -Z_{\mathrm{e}}\Gamma_{\Sigma} - \Gamma_{\delta\mathrm{H}} - [(\Gamma_{\delta\mathrm{L}} - \Gamma_{\delta\mathrm{H}})e^{-T/\tau_{\mathrm{e}}}] - \delta Z\Gamma_{\Sigma}e^{-T/\tau_{\mathrm{e}}},\\ \tfrac{dZ}{dt}(\uparrow) &= -Z_{\mathrm{g}}\Gamma_{\Sigma} - \Gamma_{\delta\mathrm{L}} - [(\Gamma_{\delta\mathrm{H}} - \Gamma_{\delta\mathrm{L}})e^{-T/\tau_{\mathrm{e}}}] - \delta Z\Gamma_{\Sigma}e^{-T/\tau_{\mathrm{e}}}.\end{aligned} \tag{S26}$$

In this case, Γ_{Σ} and $\Gamma_{\delta\delta}$ formulas are the same as (S20) and (S24), respectively. So the following analysis is the same as our FD-4 protocol. For both protocols, we first extract all $\Gamma_{\mathrm{qt},k}$-related parameters (g_k, $\Gamma_{2,k}$ and $\omega_{\mathrm{t},k}$) and Γ_{q} from the Γ_{Σ} spectrum, and then do the $\Gamma_{\delta\delta}$ spectroscopy analysis to obtain $\Gamma_{\mathrm{t},k}$ information. According to our experiment protocol, the duty cycle efficiency η is 73% for FD-4 and 50% for CD-8 (details in Table S1).

D. Choice of protocol parameters and quasi-steady state approximation

While the optimal choice of delay times in FD-4 experiment should be tailored to the qubit environment at each flux point, for convenience we used $t_{0,1,2,3} = 1$ µs, 40 µs, 150 µs, 330 µs throughout the whole spectral sweep. The choice of these delay times are motivated by the goal of sensitively detecting non-Markovian effects when the qubit and environment are both long-lived and coupled weakly to each other (i.e. the background rates away from TLS resonances) while also covering a potentially wide range of potential relaxation rates, from sub 100 µs to milliseconds. Specifically, our primary goal is to extract the initial slope $(\frac{dZ}{dt})_0$ (Eq. (S5)) reliably from the measured data at various delay times, while the accuracy of b_g and τ is of less concern. In practice, the shortest probe time t_1 is chosen based on the fastest expected decay timescale ($\sim 1/(2\Gamma_{\mathrm{qt}})$), whereas the longest probe time t_3 is selected to be long enough to capture a reasonable decay amount, for example, about 20% of the full decay contrast, under the slowest decay dynamics. We note that the long environmental response time we often observe is mainly contributed by some less-coupled but polarizable TLS such as those in the junction chain but farther-detuned, so in the spectroscopy sweep we choose a measurement time range ($T_{\mathrm{max}} \approx 20$ ms) up to a few times of expected longest TLS lifetime we can identify.

As mentioned in the main text, we apply quasi-steady approximation to TLS state within one measurement block. In this section we will also briefly discuss how qubit and TLS polarizations evolve in the FD-4

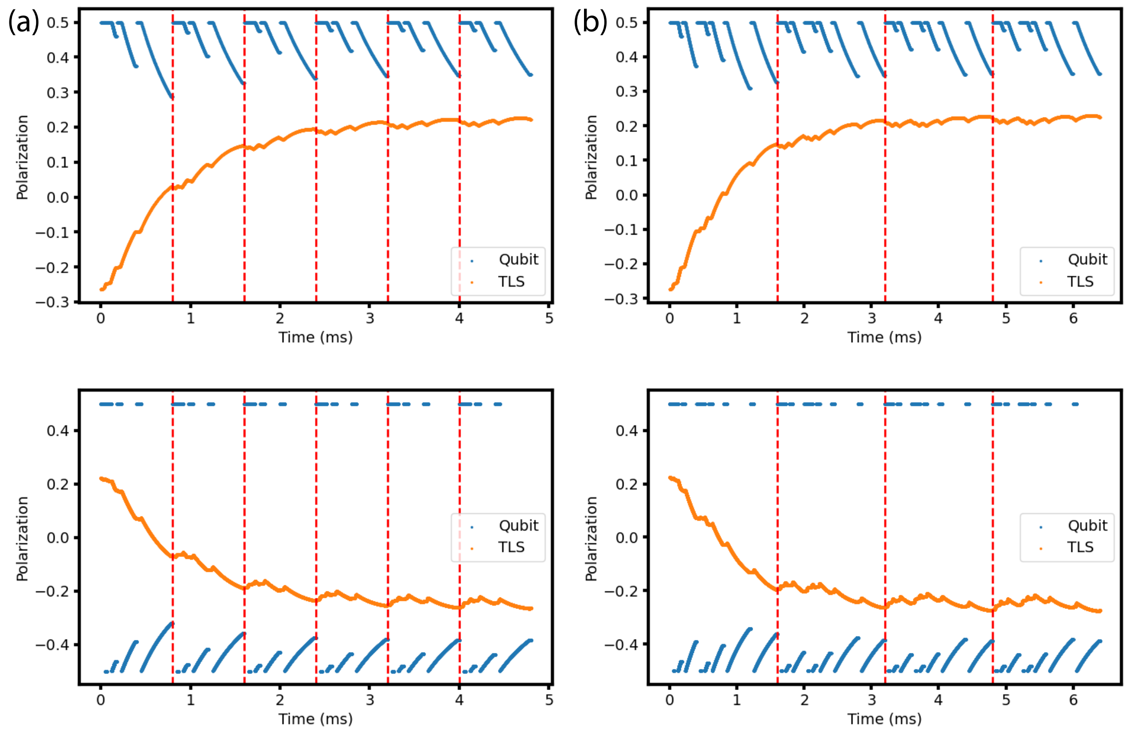

FIG. S3. **Full dynamics analysis of qubit-TLS system.** In the simulation we assume $\Gamma_{\mathrm{qt}} = 1\ \mathrm{ms}^{-1}$, $\Gamma_{\mathrm{q}} = 0.5\ \mathrm{ms}^{-1}$, $\Gamma_{\mathrm{t}} = 0.5\ \mathrm{ms}^{-1}$. Qubit is always pumped to close to $|e\rangle$ state with Z_{e} of 0.5. To initialize towards $|g\rangle$, a π_{ge} pulse is played at the end of 15 µs idle time. **(a)** Here we show qubit and TLS evolution throughout our experiment protocol till states evolution becomes stable. The blue trace represents qubit state change while the orange trace shows TLS state change. Two figures demonstrate up and down polarization process, respectively. The red vertical lines divide time by polarization sequence number, and each sequence block is 0.8 ms (detailed composition is in Table S1). **(b)** Qubit-TLS full dynamics when we move opposite readout into the closest loop. Now each polarization sequence block is 1.6 ms.

Protocol	Pumping	Idle time	t_0	t_1	t_2	t_3	Readouts	Extra waiting period	Total
FD-4	35 µs × 4	15 µs × 4	1 µs	40 µs	150 µs	330 µs	15.4 µs × 4	17.4 µs	800 µs
CD-8	35 µs × 2	15 µs × 2	Total delay time: 70 µs				15.4 µs	14.6 µs	200 µs

TABLE S1. **Time spent on each stage of two protocols.** Red parts are counted into the effective interaction portion. Extra waiting period is distributed between pulses and at the end of block measurement.

experiment. In Fig. S3 (a) we demonstrate a full dynamics of qubit-TLS evolution with some common parameters. Admittedly, the TLS state changes quite substantially during the first polarization sequence according to our protocol and it also inevitably fluctuates in the subsequent sequences, but TLS state overall follows an exponential-like pattern. In this particular run, in order to eliminate potential RO phase drifts, we build in twice

the measurements in each sequence block, leading to 1.6 ms rather than 800 μs blocks (see Section IV), which makes this approximation a bit worse (Fig. S3 (b)). To deal with stronger qubit-TLS interaction cases, we use CD-8 protocol, which was described in the Method section. Detailed time spent on each stage of two protocols is listed in Table S1.

E. Verification of methods via full dynamics simulation

Given the greater complexity of our protocols compared to a conventional T_1 measurement, we performed a self-consistency check to verify that our protocol and analysis can reliably extract the underlying qubit–TLS system parameters. Assuming that the qubit interacts with a single TLS, we specify hypothetical sets of system parameters—$\Gamma_{\rm q}$, $\Gamma_{\rm t}$, the qubit–TLS coupling strength g, and the total decoherence rate Γ_2, among others—and use full dynamical simulations of our experimental protocol to generate synthetic measurement data, including noise levels comparable to those in Fig. S2. We then apply our analysis procedure to this simulated data and compare the extracted parameters with the originally assigned ones.

In this simulation, we examine two representative cases for our 3D chip: the qubit coupled to a TLS with a millisecond-scale lifetime (2 ms) and to one with a sub-millisecond lifetime (200 μs). To further test the generality of our protocol for devices with larger $\Gamma_{\rm q}$ (such as our planar device), we vary $\Gamma_{\rm q}$ from 0.5 ms^{-1} to 5 ms^{-1}. All other parameters are kept the same across the parameter sets: both $Z^{\rm eq}$ and $p_k^{\rm eq}$ are set to -0.1

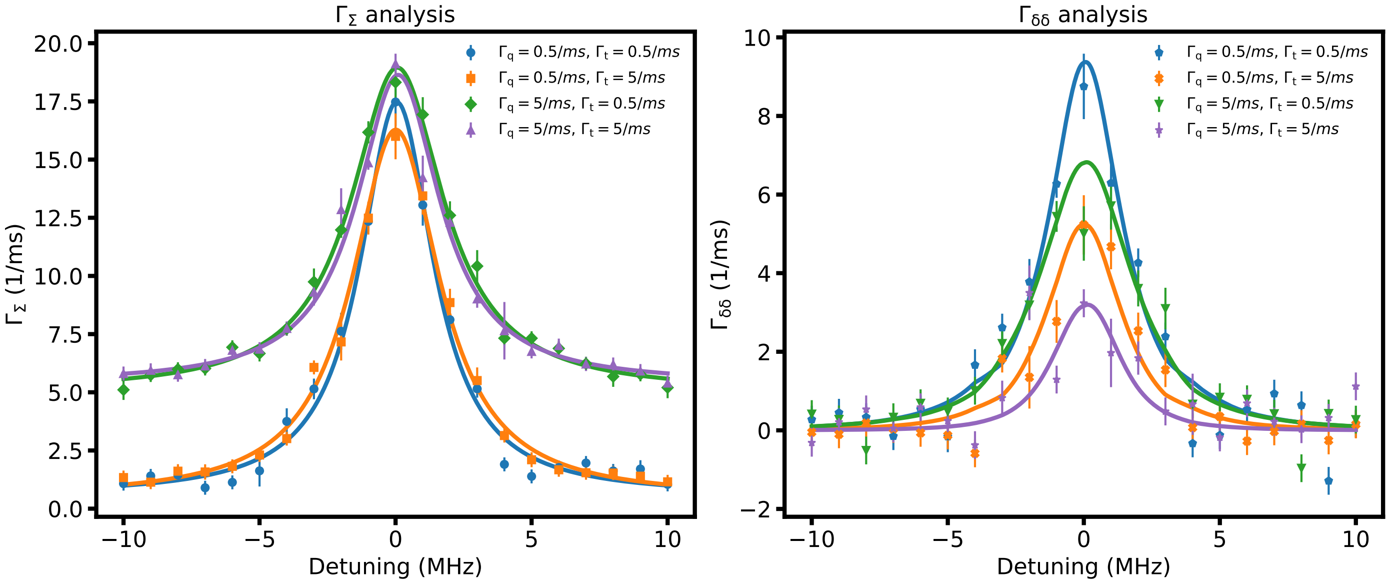

FIG. S4. **Spectroscopy analysis from the full dynamics simulation data.** Γ_Σ and $\Gamma_{\delta\delta}$ decay rates extracted from the full dynamical simulations for different combinations of $\Gamma_{\rm q}$ and $\Gamma_{\rm t}$, with same-colored curves showing their corresponding fits.

		Γ_q (ms^{-1})	Γ_t (ms^{-1})	f_TLS (MHz)	$\frac{g}{2\pi}$ (kHz)	$\frac{\Gamma_2}{2\pi}$ (MHz)
Parameter set 1	Assigned	0.5	0.5	0	50	2
	Extracted	0.55 ± 0.22	0.86 ± 0.33	0.05 ± 0.08	47 ± 2	1.6 ± 0.2
Parameter set 2	Assigned	0.5	5.0	0	50	2
	Extracted	0.45 ± 0.17	5.5 ± 1.1	0.01 ± 0.07	50 ± 1	2.0 ± 0.1
Parameter set 3	Assigned	5.0	0.5	0	50	2
	Extracted	5.0 ± 0.2	0.81 ± 0.26	0.06 ± 0.06	48 ± 1	2.1 ± 0.1
Parameter set 4	Assigned	5.0	5.0	0	50	2
	Extracted	5.4 ± 0.1	7.4 ± 1.7	0.10 ± 0.06	44 ± 1	1.9 ± 0.1

TABLE S2. **Assigned and extracted parameters from full dynamics simulation** We consider four parameter sets that vary Γ_q and Γ_t, using nominal intrinsic lifetimes of 2 ms and 200 µs as references.

(corresponding to 45% thermal population). The qubit is always pumped toward $|e\rangle$, with $Z_\mathrm{e} = 0.5$ and $Z_\mathrm{g} =$ -0.5. The TLS frequency is fixed at zero detuning, and the qubit frequency is swept from -10 MHz to +10 MHz in 1 MHz steps. For simplicity, assigned $\Gamma_\mathrm{qt} > 4\ \mathrm{ms}^{-1}$ would trigger CD-8 protocol, otherwise the data is generated by FD-4 protocol.

The spectroscopy analysis results are shown in Fig. S4 and Table. S2. We find that Γ_t tends to be slightly overestimated, primarily due to the non-negligible idle time following the reset pulse when the qubit–TLS dynamics is fast. Nevertheless, the extracted parameters show good overall agreement with the nominal values. We therefore conclude that, despite minor deviations arising from the quasi–steady-state approximation, our model reproduces the principal behavior of the qubit–TLS coupling dynamics.

IV Calibration experiments

Due to phase drift happen in our measurement from time to time, all our measurements are done in pairs: one with a qubit π_ge pulse right before readout, and one without it. The difference in the measured phase of these two readouts is recorded. To have better ability to polarize TLS and reset qubit quickly and repeatedly, we need to prepare qubit first so that they can have larger contrast between their initialization to near $|e\rangle$ and near

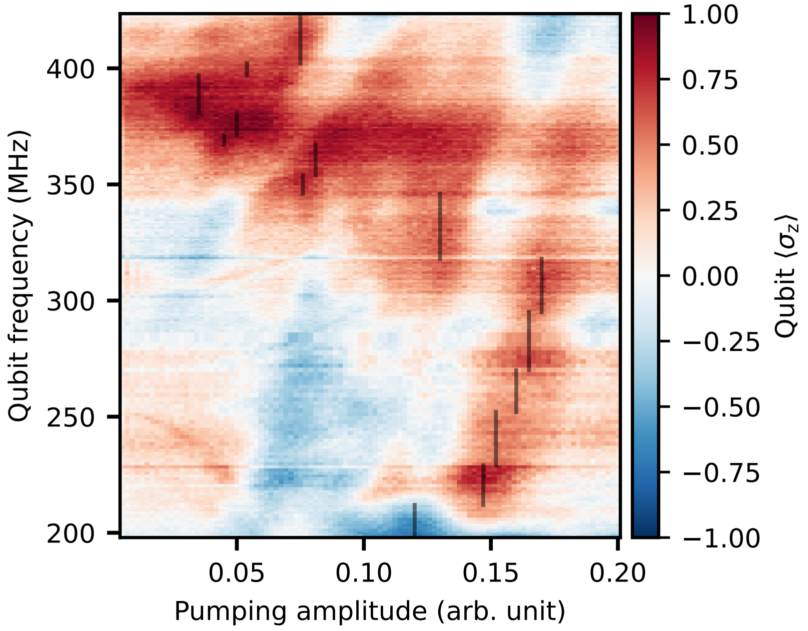

FIG. S5. **Spectroscopy sweep of measurement-induced state transition for determining reset parameters.** After a pumping pulse with various amplitude at the readout frequency, phase contrast between direct readout and readout after a π_{ge} pulse is measured, and can be converted into qubit $\langle\sigma_{\mathrm{z}}\rangle$, as shown in the color bar. Light black solid line roughly shows our chosen pumping amplitude for different frequency period.

$|g\rangle$ states, and the reset process should have minimal impact on TLS. Our qubit initialization process utilizes an effect commonly known as readout-induced state transitions, a strong readout may unconditionally pump the qubit to a specific state [S6–S8]. We apply a pumping tone at cavity frequency, followed by some idling time, to reset the qubit to near $|e\rangle$ or near $|g\rangle$ states. This process can be understood as cavity being driven into some high energy states, and swapped excitations with fluxonium's high energy states. The fluxonium then rapidly relaxes back to $|e\rangle$ or $|g\rangle$ state with a preference. After doing pumping with certain strength, phase contrast of with and without a π_{ge} pulse before the readout alters, indicating that varying pumping strength can affect qubit's final state in $|g\rangle - |e\rangle$ manifold. Choosing pumping amplitude that results in larger phase contrast can initialize our qubit well. Fig. S5 is a sample spectroscopy showing pumping power versus qubit $\langle\sigma_{\mathrm{z}}\rangle$ (processed by phase contrast and the coefficient table shown in Fig. S7) and how we choose pumping amplitude for different qubit frequencies. The very complex landscape of measurement-induced state transition implied in Fig. S5 is not well understood, but a recent study [S8] suggests that it may in fact be also related to the TLS in the device. Note that in this spectroscopy for 370 to 400 MHz, qubit initialization is high even with very low pumping amplitude, which is because previous readout plays a role in resetting the qubit.

During a readout, the ground and first excited states of the fluxonium correspond to the transmitted resonator phases $\phi_{|\mathrm{g}\rangle}$ and $\phi_{|\mathrm{e}\rangle}$, respectively (Note that our readout is probably strong enough to excite qubit to higher

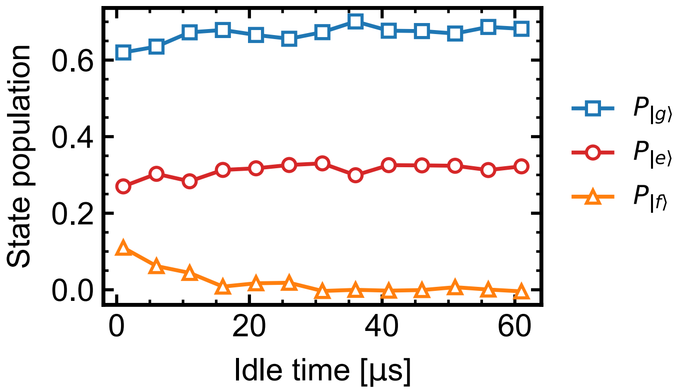

FIG. S6. **Example data of calibrated qubit population distribution following a reset pulse.** At each flux point, to calibrate our qubit reset fidelity and the $|g\rangle$-$|e\rangle$ phase contrast of our readout, we perform four different measurements following qubit reset. After we compute $\phi_{|g\rangle}$, $\phi_{|e\rangle}$ and $\phi_{|f\rangle}$ as discussed in text (Eq. (S28)), we can convert measured phase signals at different delay time into population distributions. The data shows that the reset pulse may excited the qubit to higher excited states (especially when the chosen pumping amplitudes is high), hence we incorporate a 15 µs wait time in our reset sequence to ensure that the out-of-manifold population ($P_{|f\rangle}$) is mostly eliminated.

energy state, but measuring ground and first excited states still leads to their unique transmitted resonator phases). Then with the assumption that $P_{|g\rangle} + P_{|e\rangle} = 1$ (all higher states of the fluxonium are unpopulated), we can write direct readout result M_0 as $M_0 = P_{|g\rangle}\phi_{|g\rangle} + P_{|e\rangle}\phi_{|e\rangle}$, and with a $\pi_{\rm ge}$ before readout, the result becomes M_π, where $M_\pi = P_{|e\rangle}\phi_{|g\rangle} + P_{|g\rangle}\phi_{|e\rangle}$. Now we find that:

$$M_0 - M_\pi = (P_{|e\rangle} - P_{|g\rangle})(\phi_{|e\rangle} - \phi_{|g\rangle}). \tag{S27}$$

So, as long as we know the transmitted resonator phase contrast ($\phi_{|e\rangle} - \phi_{|g\rangle}$), we can obtain $|g\rangle$ and $|e\rangle$ state populations by measuring the phase contrast of two readouts ($M_0 - M_\pi$).

As we cannot guarantee perfect qubit state preparation, we design an experiment with four measurement sequences to obtain the phase contrast ($\phi_{|e\rangle} - \phi_{|g\rangle}$). Assume that after pumping at resonator frequency, qubit consists of three states: $|g\rangle$, $|e\rangle$ and $|f\rangle$. $|f\rangle$ represents out-of-manifold state. $\phi_{|f\rangle}$ and $P_{|f\rangle}$ are the resonator phase and the population of this out-of-manifold state, respectively. All following measurements are done with different delay times after pumping. Here we list four measurement sequences:

(1) Pumping - delay - Readout,

(2) Pumping - delay - $\pi_{\rm ef}$ - Readout,

(3) Pumping - delay - $\pi_{\rm ge}$ - Readout,

(4) Pumping - delay - $\pi_{\rm ge}$ - $\pi_{\rm ef}$ - Readout.

Four measurement results M_1, M_2, M_3 and M_4 can be expressed by all states' resonator phase and popula-

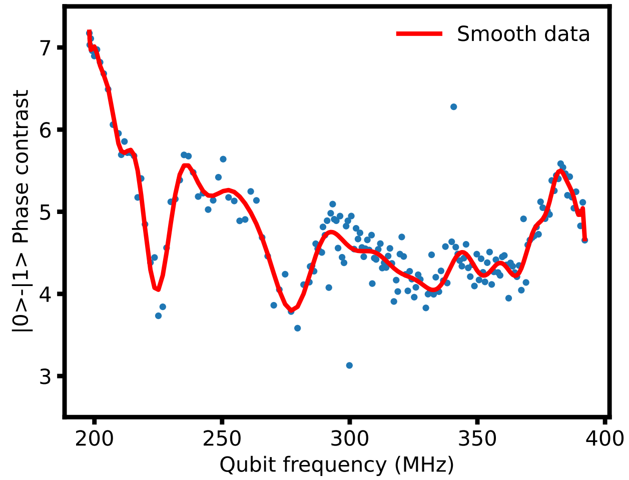

FIG. S7. **Sample data for phase-population coefficient table.** To convert phase contrast of our readouts to qubit population, we need to know what is $|g\rangle$ state to $|e\rangle$ state full phase contrast. We use a polynomial function to smooth this dataset.

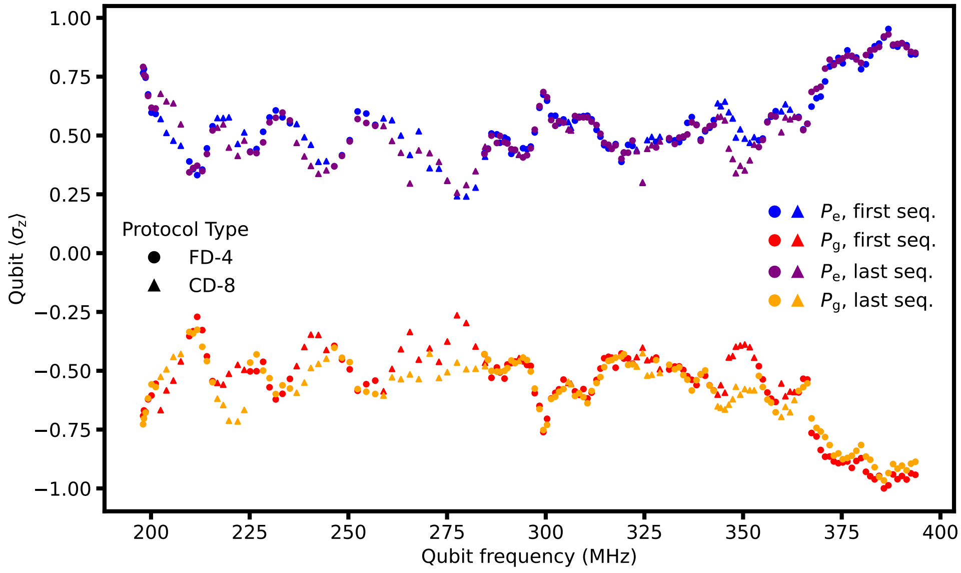

FIG. S8. **Qubit initialization performance in practice.** Red and orange data points correspond to $|e\rangle$ state initialization, while blue and purple traces show $|g\rangle$ state initialization. For FD-4 protocols, the initialization roughly stays the same for first and last sequence block and in CD-8 there is a more prominent difference which can be explained by TLS affecting qubit state during idling time.

tions, assuming all π pulses are perfect:

$$\begin{pmatrix} M_1 \\ M_2 \\ M_3 \\ M_4 \end{pmatrix} = \begin{pmatrix} P_{|\mathrm{g}\rangle} & P_{|\mathrm{e}\rangle} & P_{|\mathrm{f}\rangle} \\ P_{|\mathrm{g}\rangle} & P_{|\mathrm{f}\rangle} & P_{|\mathrm{e}\rangle} \\ P_{|\mathrm{e}\rangle} & P_{|\mathrm{g}\rangle} & P_{|\mathrm{f}\rangle} \\ P_{|\mathrm{e}\rangle} & P_{|\mathrm{f}\rangle} & P_{|\mathrm{g}\rangle} \end{pmatrix} \begin{pmatrix} \phi_{|\mathrm{g}\rangle} \\ \phi_{|\mathrm{e}\rangle} \\ \phi_{|\mathrm{f}\rangle} \end{pmatrix}. \tag{S28}$$

As shown in Fig. S6, we notice that out-of-manifold state decay fast and after a short period of time, all the readouts become stable, indicating elimination of out-of-manifold state. So we can assume that for our measurements with longest delay time, qubit does not stay in $|f\rangle$, i.e. $P_{|\mathrm{f}\rangle} = 0$, $P_{|\mathrm{g}\rangle} + P_{|\mathrm{e}\rangle} = 1$. With all these equations and assumptions, we can solve all unknown parameters and thus know the resonator phase contrast when qubit is at $|g\rangle$ and $|e\rangle$, $\phi_{|\mathrm{e}\rangle} - \phi_{|\mathrm{g}\rangle}$. Data shows that generally if we apply 15 µs idle time after pumping, out-of-manifold qubit state can be mostly back to $|g\rangle$ and $|e\rangle$ manifold. Fig. S7 provides the calculated ($\phi_{|\mathrm{e}\rangle} - \phi_{|\mathrm{g}\rangle}$) phase contrast versus qubit frequency for the spectroscopy sweep in Fig. 4(c) in main text. Fig. S8 demonstrates how our pumping strategy performs in the experiments that produce spectroscopy shown in main text. Data points above 270 MHz are obtained by averaging the forward and backward runs, while those below 270 MHz are taken from the second run.

V Two-timescale relaxometry with variable polarity-switch structure

Separate from FD-4 and CD-8 experiments we discussed in the main text, we also proposed an experiment to directly probe how long the qubit can memorize measurement history due to the TLS bath (we will call it "polarity switch" for short). As shown in Fig. S9, this experiment simply uses same delay time and repeats polarizing TLS in one direction for a few times before the direction is reversed. If the TLS bath is completely not polarizable at this qubit frequency, i.e. qubit state only depends on current sequence and does not have any memory effect, then all readouts followed by same qubit initialization direction should be equal. Otherwise, there would be a memory time scale for each polarizable TLS, showing how much they can be affected by qubit's polarizing effect happened some sequences before. In the sequences we applied to obtain spectroscopy, we gradually increased number of one-direction polarizing sequence to observe short and long memory effect at the same time.

The analysis is done in the following way. First we consider qubit coupled to one TLS. Qubit and TLS states after n^{th} delay depend on their states after $(\mathrm{n}-1)^{\mathrm{th}}$ delay, following the Solomon equations:

$$\frac{dZ}{dt} = -\Gamma_{\mathrm{q}}(Z - Z^{\mathrm{eq}}) - \Gamma_{\mathrm{qt}}(Z - P_{\mathrm{t}}), \tag{S29}$$

$$\frac{dP_{\mathrm{t}}}{dt} = -\Gamma_{\mathrm{t}}(P_{\mathrm{t}} - P_{\mathrm{t}}^{\mathrm{eq}}) - \Gamma_{\mathrm{qt}}(P_{\mathrm{t}} - Z). \tag{S30}$$

This differential equation set can be solved as:

$$\begin{pmatrix} Z_n \\ P_{\mathrm{t},n} \end{pmatrix} = e^{[\Gamma]\cdot t} \cdot \left\{ \begin{pmatrix} R_n \\ P_{\mathrm{t},n-1} \end{pmatrix} + [\Gamma]^{-1} \cdot \begin{pmatrix} \Gamma_\mathrm{q} \cdot Z^{\mathrm{eq}} \\ \Gamma_\mathrm{t} \cdot P_\mathrm{t}^{\mathrm{eq}} \end{pmatrix} \right\} - [\Gamma]^{-1} \cdot \begin{pmatrix} \Gamma_\mathrm{q} \cdot Z^{\mathrm{eq}} \\ \Gamma_\mathrm{t} \cdot P_\mathrm{t}^{\mathrm{eq}} \end{pmatrix}, \tag{S31}$$

in which Z_n is the qubit state readout in the nth sequence, $P_{\mathrm{t},n}$ represents the TLS state after n^{th} delay, R_n is the qubit state preparation for n^{th} sequence, and

$$[\Gamma] = \begin{bmatrix} -\Gamma_\mathrm{q} - \Gamma_\mathrm{qt} & \Gamma_\mathrm{qt} \\ \Gamma_\mathrm{qt} & -\Gamma_\mathrm{t} - \Gamma_\mathrm{qt} \end{bmatrix}. \tag{S32}$$

For multiple TLS cases, we assume different TLS don't have direct coupling (they can still couple through the fluxonium qubit). $P_{\mathrm{t},n}$ can be replaced by $\mathbf{P}_{\mathrm{t},n}$, and so are the Γ_t and $P_\mathrm{t}^{\mathrm{eq}}$ terms. $[\Gamma]$ becomes:

$$\begin{bmatrix} -\Gamma_\mathrm{q} - \sum_i \Gamma_{\mathrm{qt},i} & \Gamma_{\mathrm{qt},1} & \Gamma_{\mathrm{qt},2} & \cdots & \Gamma_{\mathrm{qt},k} \\ \Gamma_{\mathrm{qt},1} & -\Gamma_{\mathrm{t},1} - \Gamma_{\mathrm{qt},1} & 0 & \cdots & 0 \\ \Gamma_{\mathrm{qt},2} & 0 & -\Gamma_{\mathrm{t},2} - \Gamma_{\mathrm{qt},2} & \cdots & 0 \\ \vdots & \vdots & \vdots & \ddots & \vdots \\ \Gamma_{\mathrm{qt},k} & 0 & 0 & \cdots & -\Gamma_{\mathrm{t},k} - \Gamma_{\mathrm{qt},k} \end{bmatrix}. \tag{S33}$$

We can rewrite the formula by letting $e^{[\Gamma]\cdot t} = \begin{pmatrix} T_{\mathrm{qq}} & \mathbf{T}_{\mathrm{qt}}^T \\ \mathbf{T}_{\mathrm{qt}} & [T_{\mathrm{tt}}] \end{pmatrix}$, and combine all terms related to equilibrium populations together:

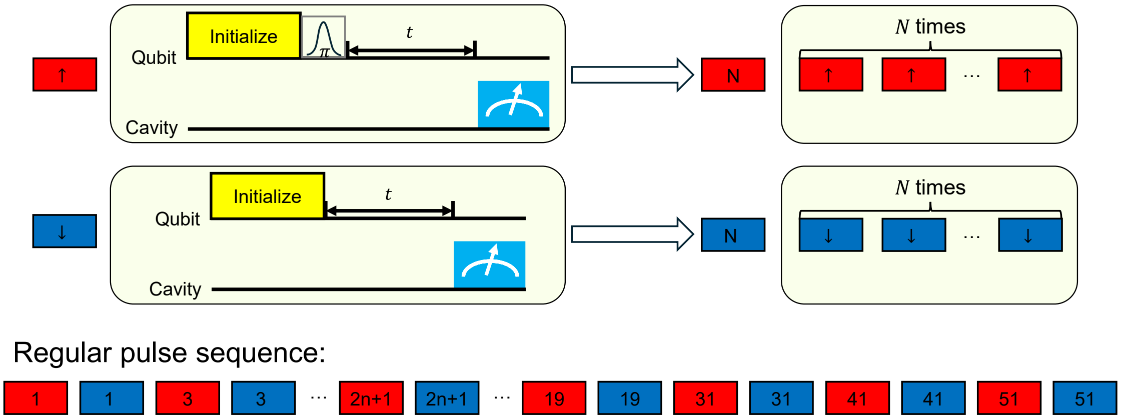

FIG. S9. **Pulse sequence diagram for polarity switch** Polarity switch experiment consists of a number of continuous "qubit initialization–delay–measure" sequences. The "↓" initialization requires only pumping, while the "↑" initialization requires pumping followed by an additional π pulse. The whole pulse sequence can be arbitrary long continuous measurements. For the spectroscopy study, We use an alternating sequence protocol that can probe both short and long memory effect.

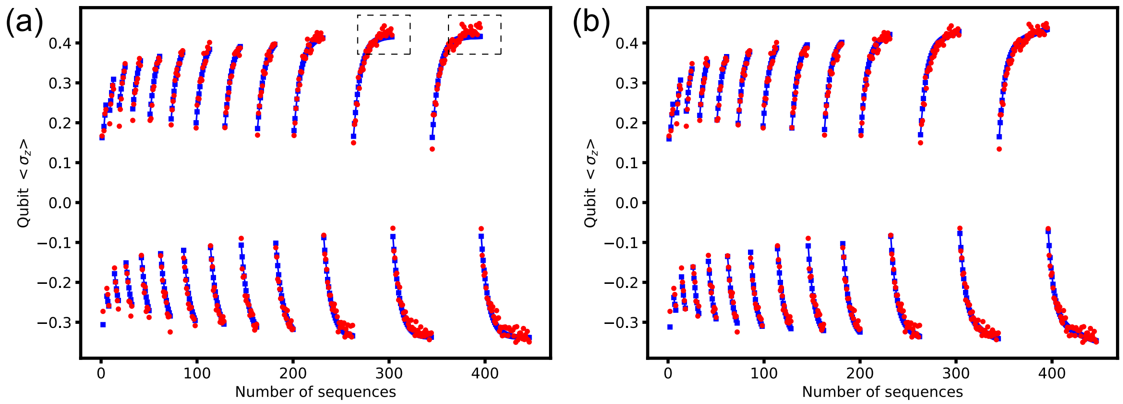

FIG. S10. **Example relaxometry data with polarity switch.** Here we show a sample data (red scatter points) for polarity switch experiment with two fittings in blue lines. For plot (a) we assume one TLS timescale and for plot (b) we apply two. Experiment sequence is shown in Fig. S9, delay time t = 130 µs. We believe qubit is affected by at least two TLS time scales as one time scale fitting fails to track qubit population tails of long polarizing sequences (see parts enclosed by the dotted line in (a)). Among two timescales, one is for a relatively strong local TLS, and the other for a background TLS bath effect. Two timescale exponential moving average model shows that here the shorter memory time (n_1) is 6 sequences and the background memory time (n_2) is 115 sequences.

$$\begin{pmatrix} Z_n \\ \mathbf{P}_{\mathrm{t},n} \end{pmatrix} = \begin{pmatrix} T_{\mathrm{qq}} & \mathbf{T}_{\mathrm{qt}}^T \\ \mathbf{T}_{\mathrm{qt}} & [T_{\mathrm{tt}}] \end{pmatrix} \begin{pmatrix} R_n \\ \mathbf{P}_{\mathrm{t},n-1} \end{pmatrix} + \begin{pmatrix} Z^{\mathrm{th}} \\ \mathbf{P}_{\mathrm{t}}^{\mathrm{th}} \end{pmatrix}. \tag{S34}$$

- $\mathbf{P}_{\mathrm{t},n}$ and $\mathbf{P}_{\mathrm{t},n-1}$ are the TLS states after n^{th} and $(\mathrm{n}-1)^{\mathrm{th}}$ sequence, respectively.
- $\begin{pmatrix} T_{\mathrm{qq}} & \mathbf{T}_{\mathrm{qt}}^T \\ \mathbf{T}_{\mathrm{qt}} & [T_{\mathrm{tt}}] \end{pmatrix}$ is the evolution matrix.
- Z^{th} and $\mathbf{P}_{\mathrm{t}}^{\mathrm{th}}$ are some parameters related to the thermal equilibrium conditions about qubit and TLS, respectively.

Use this recurrence relation to expand $\mathbf{P}_{\mathrm{t},n-1}$, finally we can get formula about Z_n:

$$Z_n = T_{\mathrm{qq}} R_n + \mathbf{T}_{\mathrm{qt}}^T \mathbf{T}_{\mathrm{qt}} R_{n-1} + \mathbf{T}_{\mathrm{qt}}^T [T_{\mathrm{tt}}] \mathbf{T}_{\mathrm{qt}} R_{n-2} + \mathbf{T}_{\mathrm{qt}}^T [T_{\mathrm{tt}}]^2 \mathbf{T}_{\mathrm{qt}} R_{n-3} + \ldots + C, \tag{S35}$$

where C is a constant term recording the qubit and TLS thermal equilibrium information.

Now we can diagonalize $[T_{\mathrm{tt}}]$, and the transformation matrix can be absorbed by $\mathbf{T}_{\mathrm{qt}}^T$ and $\mathbf{T}_{\mathrm{qt}}$. Thus, each element in $[T_{\mathrm{tt}}]$ matrix's diagonal corresponds to an exponential moving average term.

Finally we can write:

$$Z_n = T_{\mathrm{qq}} R_n + C + \sum_{i=0}^{\infty} \sum_k e^{-i/n_k} R_{n-i} C_k, \tag{S36}$$

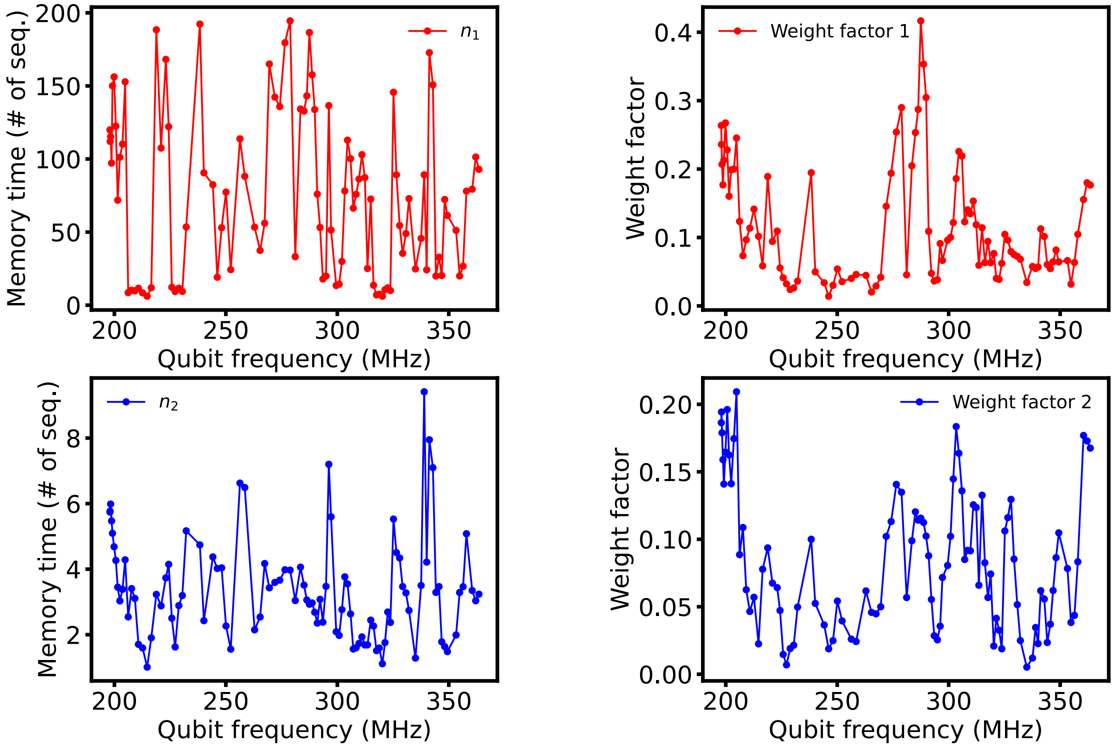

FIG. S11. **Polarity switch experiment spectroscopy.** Sample spectroscopy for polarity switch experiment contains each TLS's memory time (n_1, n_2) and weight factors (n_1C_1, n_2C_2). The total duration of each sequence is 200 µs. In this analysis we assume that fluxonium at any frequency is always influenced by two environment memory time scales. Generally qubit is affected by strongest neighboring TLS with a memory time of less than 10 sequences (n_2) and a background TLS bath effect with scores of sequences memory time (n_1). But there are also cases qubit being affected by two strong TLS simultaneously (as shown in very low n_1 cases).

where sum of i is to add up memory effect contributed by all the previous sequences, and k sums up all TLS's contributions. C_k is a constant factor for each TLS.

In Fig. S10 we show a sample dataset applying analysis above. Besides the short memory time scale of 6 sequences, we find clue of a much longer memory time scale (115 sequences) when we focus on the tails of long polarizing sequences. Fig. S11 shows a spectroscopy sweep result. We apply same sequence shown in Fig. S9 with t = 130 µs to qubit frequency ranging from 200 to 360 MHz. We note that generally $C_2 \gg C_1$ does not necessarily mean short memory time scale contributes more to current status than the longer one, as long memory time makes earlier sequences more important. So the weight factor shown in the right panel is defined as n_kC_k, which can be understood as the coefficient of the moving average term's integral. These very long memory effects may be hidden in the two-time-scale relaxometry methods due to data resolution.

VI Surface participation ratio (SPR) simulation and TLS electric dipole moment

A. SPR simulation

In this sub-section we discuss our SPR simulation method. Like [S9] that applied two-step simulations for transmon qubits, we do simulation for the full 3D fluxonium qubit in a commercial high-frequency electromagnetic solver (Ansys HFSS), aiming to solve electric field distribution far from Josephson junction parts, and use low-frequency electromagnetic simulation software (Ansys Maxwell) to extract electric field information close to Josephson junctions. Here we give more detailed information for metal-substrate (MS) interface SPR and note total SPR for fluxonium substrate-air (SA) interface is roughly 3.6×10^{-4} excluding regions close to small junction. Metal-air (MA) SPR is small compared to MS. Except MS, we further take dielectric layer inside Josephson junctions into account.

Fig. S12 and its subfigure show the qubit part in global and local simulation, respectively. In the global simulation, we display the whole fluxonium qubit but only take information for large leads (in blue) and pads (in purple). The small junction is represented by one lumped element, and the junction chain is in the form of ten lumped elements, each of which stands for 16.6 large junctions in the chain with integrated capacitance and inductance value. Applying multiple lumped elements to represent junction chain is to better imitate junction

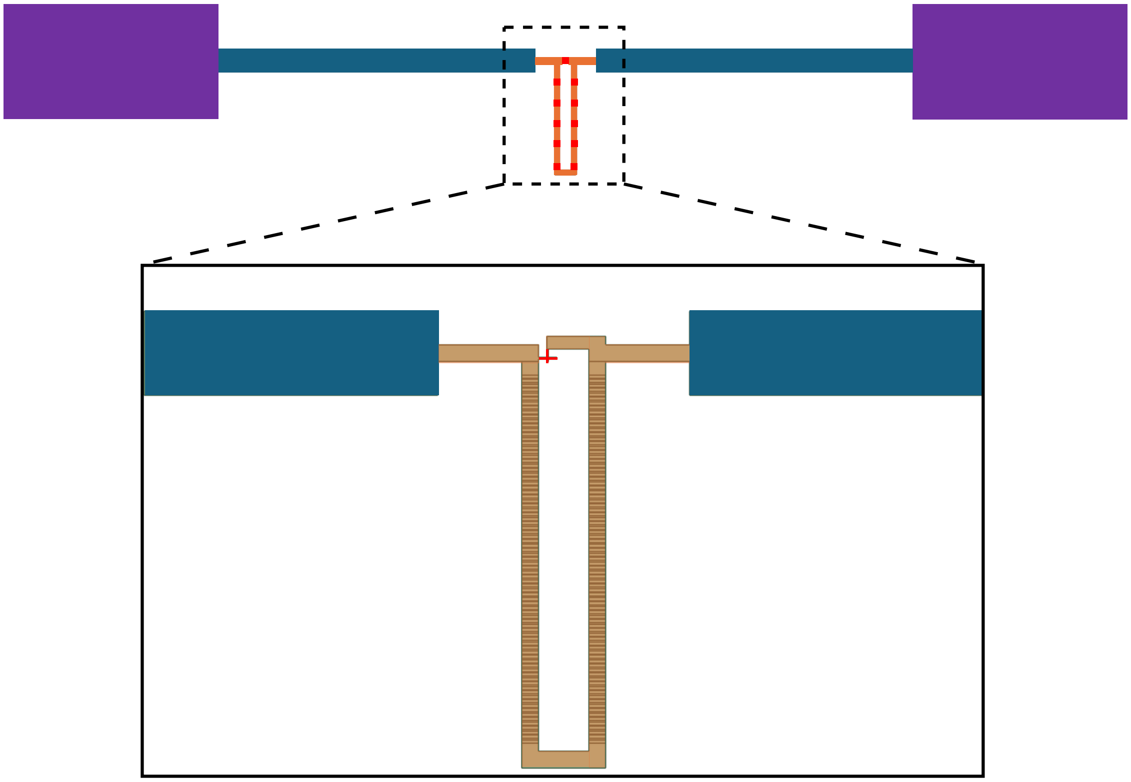

FIG. S12. **Fluxonium qubit in global (Ansys HFSS) and local (Ansys Maxwell) simulations**

	MS pads	MS leads			Small junction	Junction chains
		far from junctions	under junction chain	near small junction(s)		
Fluxonium	0.78	2.1	1.3	18.7	1160	200
Transmon	0.79	0.15	/	4.3	651	/

TABLE S3. **Total SPR in parts for fluxonium and tunable transmon device.** All participation ratio results need to further multiplied by 10^{-4}. For the fluxonium device, as shown in Fig. S12, "MS pads" simply refer to purple parts, and "MS leads near small junction" include the red cross pattern for small junction. Dielectric layer right under the junction chain falls into the category of "MS leads under junction chain". The rest of leads, including dark blue parts and parts of brown area, are classified into "MS leads far from junctions". As for transmon, dividing point between near and far from junctions is 1 μm to two Josephson junctions.

chain's electric field in global simulation. In local simulation we draw out details of junction chain and small junction, and focus on obtaining electric field distribution for junction array (in brown) and the small junction (in red). We assume a 1.75 nm barrier thickness for all Josephson junction in this fluxonium qubit (transmon's barrier thickness is assumed to be 1.5 nm due to much larger critical current density J_c). Electric field results in two simulations are related to each other by normalizing electric field energy inside the small junction, i.e. the transfer coefficient factor:

$$F^2 = \frac{U_{j,\mathrm{HFSS}}}{U_{j,\mathrm{Maxwell}}} = \frac{C_l V_l^2}{2\int_V U dV} = \frac{C_l\left(\int_0^L E_{//}\, dl\right)^2}{\int_V \varepsilon_0\varepsilon_r E^2\, dx\, dy\, dz},$$

$E_{//}$ is parallel electric field along lumped element's middle line and C_l is lumped element capacitance. Denominator includes energy volume integral to the small junction dielectric layer.

In table S3 we show a tunable transmon [S10] and 3D fluxonium device's total SPR numbers for different parts. Note that the first three regions involve large physical volumes, so dielectric loss from TLSs in these areas can be modeled using a uniform loss tangent, which produces a background decay rate. The remaining three regions, by contrast, have very large participation ratios. However, TLSs in these regions may either be absent in the frequency window of our spectroscopy by chance, or they may appear as discrete spectral peaks.

B. TLS dipole moment estimation

According to our experience to discrete TLS analysis, qubit-TLS coupling $g/(2\pi)$ ranges from 20 kHz to 80 kHz, and average value is around 55 kHz. The electric field inside small junction of fluxonium is derived as $\widehat{E} = \frac{\widehat{Q}}{Cd} = \frac{4E_\mathrm{c}\widehat{n}}{e_0 d}$, where e_0 is elementary charge and d is junction oxide layer thickness (1.75 nm as assumed before). Therefore, the oscillation field strength $|E|$ = 630 V/m. An crude estimation of average electric field

strength in the junction chain is $|E_0| = \frac{|E|}{n_0}$ = 3.8 V/m, where n_0 is the number of junctions in the junction chain. When we consider mutual capacitance between all metal pieces, the result shows that $|E_0|$ ranges from 1.5 V/m to 11 V/m. Using $|E_0| = 3.8$ V/m, we estimate average effective TLS dipole of $p_z \cos\theta = 2\hbar g/E_0 = 1.2$ eÅ, where p_z is the electric dipole moment along the direction of the field, and $\theta = \arctan\frac{\epsilon}{\Delta}$ is the TLS mixing angle between the asymmetry energy and tunneling energy.

[S1] R. Barends, J. Kelly, A. Megrant, D. Sank, E. Jeffrey, Y. Chen, Y. Yin, B. Chiaro, J. Mutus, C. Neill, P. O'Malley, P. Roushan, J. Wenner, T. C. White, A. N. Cleland, and J. M. Martinis, Coherent Josephson Qubit Suitable for Scalable Quantum Integrated Circuits, Physical Review Letters **111**, 080502 (2013).

[S2] I. Solomon, Relaxation Processes in a System of Two Spins, Physical Review **99**, 559 (1955).

[S3] M. Spiecker, P. Paluch, N. Gosling, N. Drucker, S. Matityahu, D. Gusenkova, S. Günzler, D. Rieger, I. Takmakov, F. Valenti, P. Winkel, R. Gebauer, O. Sander, G. Catelani, A. Shnirman, A. V. Ustinov, W. Wernsdorfer, Y. Cohen, and I. M. Pop, Two-level system hyperpolarization using a quantum Szilard engine, Nature Physics **19**, 1320 (2023).

[S4] M. Odeh, K. Godeneli, E. Li, R. Tangirala, H. Zhou, X. Zhang, Z.-H. Zhang, and A. Sipahigil, Non-Markovian dynamics of a superconducting qubit in a phononic bandgap, Nature Physics **21**, 406 (2025).

[S5] M. Spiecker, A. I. Pavlov, A. Shnirman, and I. M. Pop, Solomon equations for qubit and two-level systems: Insights into non-Poissonian quantum jumps, Physical Review A **109**, 052218 (2024).

[S6] D. Sank, Z. Chen, M. Khezri, J. Kelly, R. Barends, B. Campbell, Y. Chen, B. Chiaro, A. Dunsworth, A. Fowler, E. Jeffrey, E. Lucero, A. Megrant, J. Mutus, M. Neeley, C. Neill, P. O'Malley, C. Quintana, P. Roushan, A. Vainsencher, T. White, J. Wenner, A. N. Korotkov, and J. M. Martinis, Measurement-Induced State Transitions in a Superconducting Qubit: Beyond the Rotating Wave Approximation, Physical Review Letters **117**, 190503 (2016).

[S7] M. F. Dumas, B. Groleau-Paré, A. McDonald, M. H. Muñoz-Arias, C. Lledó, B. D'Anjou, and A. Blais, Measurement-Induced Transmon Ionization, Physical Review X **14**, 041023 (2024).

[S8] A. Bista, M. Thibodeau, K. Nie, K. Chow, B. K. Clark, and A. Kou, Readout-induced leakage of the fluxonium qubit (2025), arXiv:2501.17807 [quant-ph].

[S9] C. Wang, C. Axline, Y. Y. Gao, T. Brecht, Y. Chu, L. Frunzio, M. H. Devoret, and R. J. Schoelkopf, Surface participation and dielectric loss in superconducting qubits, Applied Physics Letters **107**, 162601 (2015).

[S10] B.-J. Liu, Y.-Y. Wang, T. Sheffer, and C. Wang, Observation of Discrete Charge States of a Coherent Two-Level System in a Superconducting Qubit, Physical Review Letters **133**, 160602 (2024).
